# Analysis of the factors affecting the quality of life in patients with pituitary adenomas before and after endoscopic transsphenoidal surgery

**DOI:** 10.3389/fendo.2026.1796777

**Published:** 2026-08-13

**Authors:** Wanlin Liang, Xun Xia, Kexia Fan, Tao Yang, Jingmin Cheng, Xiansong Zhu, Yunxing Li, Jie Wang, Yuan Ma, Yongxiang Yang

**Affiliations:** 1 Department of Neurosurgery, The General Hospital of Western Theater Command, Chengdu, China; 2 School of Clinical Medicine, Chengdu Medical College, Chengdu, China; 3 College of Medicine, Southwest Jiaotong University, Chengdu, China; 4 The Affiliated Hospital of Southwest Medical University, Luzhou, China

**Keywords:** pituitary adenomas, quality of life, ETSS, vision, headache, anxiety or depression

## Abstract

**Objective:**

Pituitary adenomas (PAs) not only cause physiological symptoms, but also lead to psychological symptoms, which result in the decline of the quality of life (QOL) ultimately. This study aims to identify the factors that might influence the QOL of patients with PAs before and after endoscopic transsphenoidal surgery (ETSS).

**Methods:**

A total of 83 patients with PAs who underwent ETSS and completed the visual-related QOL (VR-QOL), headache impact test (HIT-6), Hamilton anxiety/depression rating scale (HAMA/HAMD), and 36-items health-related QOL (HR-QOL) questionnaires preoperatively and at 1/3 months postoperatively were recruited. According to the preoperative physiological/psychological HR-QOL, they were divided into four groups. And, the factors that might be correlated with the impairment of physical/psychological QOL before ETSS and the improvement of physical/psychological QOL after ETSS were identified by comparative and regressive analysis in terms of gender, age, tumor size, pathological type, VR-QOL, HIT-6, HAMA and HAMD.

**Results:**

As for the patients with PAs before ETSS, the degree of physiological QOL impairment was positively correlated with VR-QOL and was negatively correlated with HIT-6 and HAMD score, while the degree of psychological QOL impairment was positively correlated with age and was negatively correlated with HIT-6, HAMA and HAMD score. After those patients underwent ETSS, they gradually acquired the improvements of physiological and psychological QOL from 1 to 3 months, which were positively correlated with the increased value of VR-QOL and the decreased value of HIT-6, HAMA and HAMD scores.

**Conclusions:**

The impairment of HR-QOL before ETSS and the improvement of HR-QOL after ETSS in patients with PAs were closely correlated with headache, subjective perception of vision and visual field loss, and the anxiety/depression emotions.

## 1 Introduction

Pituitary adenomas (PAs) are the most common intracranial tumors in the sellar region, with an overall estimated prevalence of 17% more or less based on radiologic and autopsy studies (1, 2). PAs not only cause physiological symptoms such as headache or visual disturbance, but also lead to psychological symptoms such as higher anxiety/depression-related personality traits, which result in a decline in the quality of life (QOL) ultimately (3). At present, the most effective and widely used treatment for PAs patients is endoscopic transsphenoidal surgery (ETSS), which has the advantages including small surgical trauma, close observation of the anatomical structure around tumor, high rate of total tumor resection, low probability of nerve and vascular injury, and short hospital stays (3–5). The above superiorities of ETSS suggest that this treatment may exert benefit on the postoperative QOL of patients with PAs, although this hypothesis and the potential mechanisms have not yet been clearly elucidated. In order to clarify this issue preliminary, we conducted this prospective observational clinical study to identify the key factors that can influence the QOL of patients with PAs before and after ETSS.

PAs often result in the impairment of QOL in terms of physical and mental aspects, attributed to the space-occupying and endocrine effects such as the compression of optic nerve and the hormone hypersecretion or hypopituitarism. Headache in patients with PAs is correlated with tumor volume, the relationship with sellar structures, functional disturbance of the hypothalamo- pituitary axis, patient predisposition and family history, which may be an incapacitating symptom by impacting QOL and daily life (6–8). PAs can cause compression effects on the optic nerve and optic chiasm, often leading to vision-related symptoms such as visual field defects, visual acuity changes or ophthalmoplegia, and further impairing the vision-specific QOL of patients (9). In addition to headache and vision loss, patients with PAs may also experience pituitary dysfunction or systemic endocrine changes such as hypopituitarism, hyperprolactinemia, Cushing’s syndrome or hyperthyroidism and so on (3, 10, 11). The endocrine consequences of PAs not only lead to physiological dysfunctions such as insomnia and fatigue, but also cause anxiety/depression-related personality traits changes, which further impair the physical/mental aspects of QOL (3, 12). Among them, hypopituitarism is a clinical syndrome caused by the inadequate secretion of one or more anterior/posterior pituitary hormones, which can influence the QOL of PAs patients preoperatively/postoperatively (4, 13). Taking together, PAs can affect patients’ QOL in many aspects, and it is in urgent need to explore more reasonable and effective measures to improve the QOL of these patients.

ETSS is the most effective surgical treatment for PAs, which is associated with significant higher rate of symptom relief, shorter hospital stay, fewer complications, and higher survival rate (5, 14). Recently, several studies have demonstrated that patients with PAs underwent ETSS acquired QOL benefit including higher physical and mental score post operation (14, 15). In order to deeply analyze the key factors influencing the QOL of patients with PAs before and after surgery, and further to explore potential interventional factors to improve the QOL of patients to a greater extent, we conducted this prospective study.

## 2 Materials and methods

### 2.1 Setting

This prospective and observational clinical study was conducted in China at The General Hospital of Western Theater Command in Chengdu, which has a professional multidisciplinary diagnosis and treatment team for PAs. This team was consisted of professional doctors from the department of neurosurgery, endocrinology, otolaryngology and radiology. This study strictly adhered to the ethical principles listed in the Declaration of Helsinki and obtained ethical approval from the Ethics Committee of The General Hospital of Western Theater Command. All the studying processes in this research were carried out in accordance with the approved guidelines.

### 2.2 Patients

All adult patients who were diagnosed with PAs and underwent ETSS for tumor resection in our institution from October 2023 to December 2024 were enrolled in the study. All the patients wrote informed consent prior to participation. The diagnosis of PAs was determined on the basis of imaging manifestations including computed tomography (CT) and magnetic resonance imaging (MRI), and endocrinological indicators. The inclusion criteria including: (1) Age≥18 years; (2) Admission diagnosis was PAs;(3) Having the indications for ETSS; (4) The treatment method chosen by the patient and their family members was ETSS; (5) Completed the proposed schedule of questionnaire survey voluntarily. The exclusion criteria including: (1) Without the indications for ETSS; (2) The treatment method chosen by the patient and their family members was medication therapy or craniotomy; (3) Unable or unwilling to complete the proposed schedule of questionnaire survey; (4) Pre-existing other diseases other than PAs including cardiovascular system diseases, respiratory system diseases, digestive system diseases, nervous system diseases, musculoskeletal diseases, immune system diseases, infectious diseases, and so on before the ETSS; (4) Presence of complications including cerebrospinal fluid leakage, intracranial infection, neurovascular injury, pituitary dysfunction, and so on after the ETSS.

### 2.3 Preoperative evaluation

Once patients arrived at our department, standard treatments and managements were carried out according to the Diagnosis and Treatment Guidelines for PAs (16). All recruited patients received comprehensive neurological evaluation, MRI and CT scan, ophthalmic examination (visual acuity and visual field testing) and endocrinological evaluation. And, other routine clinical examinations including chest X-Ray, abdomen ultrasound, electrocardiogram and laboratory tests including hematology, urine and feces analysis, lipid and coagulation profile, multiorgan (cardiac, liver, renal) function analysis were conducted as well. According to the MRI and endocrinological evaluation, PAs were classified into microadenomas (<10mm)/macroadenomas (≥10 mm)/giant adenomas (>30mm), secreting/non-secreting tumors, and Knosp Grade 1–4 reflecting parasellar invasion and suprasellar extension. The diagnostic criteria for hypopituitarism before operation are made according to the endocrine tests and clinical symptoms. Patients with adrenal axis insufficiency manifest as fatigue, hypotension and hypoglycemia, the morning cortisol level <3 ug/dL, and the insulin hypoglycemia test or ACTH stimulation test shows the cortisol peak <18 ug/dL. Patients with thyroid axis insufficiency manifest as cold intolerance, weight gain and sluggishness, and the free T4 and TSH is below the lower limit of the normal range. Patients with gonadal axis insufficiency manifest as amenorrhea in women and infertility in men, low estradiol or testosterone. Patients with growth hormone axis insufficiency manifest as slow growth in children, fatigue and weakness in adults, IGF-1 is lower than the normal value of the same age group, the insulin hypoglycemia test or arginine-GHRH test shows the GH peak <5.1 ng/mL. The treatment plans and ETSS indications for PAs were jointly formulated by a multidisciplinary diagnosis and treatment team including a neurosurgeon, an endocrinologist, an otolaryngologist, and a radiologist. If the patient has indications of ETSS and both the family members and the patient voluntarily chooses surgical treatment, the ETSS would be carried out.

### 2.4 Surgical procedures of ETSS

Before the operation, professional neuroendoscopist and otolaryngologist firstly identified the adjacent relationship between the tumor and the nerves/blood vessels, and the anatomical structure of the sphenoid sinus and sellar floor bone by MRI and CT image. Then, the ETSS was performed via an expanded endoscopic approach in all patients by the same professional neuroendoscopist. The surgical procedures of ETSS were elaborated as below. First, the middle and superior turbinate were slightly pushed outward, an incision was made and a part of the nasal septal mucosal flap was retained to expose the anterior wall of the sphenoid sinus. Secondly, the anterior wall bone of the sphenoid sinus was grounded off by a grinding drill to enter the sphenoid sinus cavity and expose the bone structure of the sellar floor fully. Then, the bone at the base of the sella turcica was grinded off, and the dura mater at the base of the sella turcica, the internal carotid artery and the wall of the cavernous sinus were fully exposed. Finally, the dura mater was incised to fully expose and remove the tumor. At last, multiple layers of repair were carried out using autologous mucosa, autologous fat, autologous fibrous glue and nasal septum mucosal flap.

### 2.5 The pathological classification of PAs

The pathological diagnosis of pituitary adenomas is made based on the latest classification criteria of the World Health Organization (WHO) in 2022. This diagnostic standard no longer solely considers the type of hormones secreted by the tumor, but first determines the “cell lineage” source of the tumor through transcription factors, and then combines hormones and other markers for precise classification. In this study, the pathological diagnosis first determines which pituitary cell lineage the tumor originates from by detecting three main transcription factors through immunohistochemistry, such as PIT1 corresponding to the growth hormone prolactin and thyroid stimulating hormone lineages, TPIT corresponding to the adrenocorticotropic hormone lineage, and SF1 corresponding to the gonadotropin lineage. Then, the actual secreted hormones of the tumor cells (such as GH, PRL, ACTH, etc.) were detected to clarify their functionality, and the cell keratin and other markers were examined to assist the classifications of different subtypes within the same lineage (such as dense granular and sparse granular growth hormone tumors). If all the transcription factors and hormones are negative, and other tumors (such as paragangliomas) are excluded, then the diagnosis is zero-cell adenoma.

### 2.6 Questionnaires

All the recruited patients who were diagnosed with PAs and underwent ETSS had completed the Visual-related QOL (VR-QOL), Headache Impact Test (HIT-6), Hamilton Anxiety/Depression Rating Scale (HAMA/HAMD), and 36-item Health-related QOL (HR-QOL) questionnaire preoperatively and at 1/3 months postoperatively.

#### 2.6.1 Visual-related quality of life

Visual-related Quality of Life (VR-QOL) was measured by the National Eye Institute 25-Item Visual Function Questionnaire (NEI-VFQ-25), which is consisted of 25 questions based on visual function and subjective well-being of PAs patients. Those 25 questions are categorized into 12 subscales including general-health status (question 1), general vision (question 2-3), ocular pain (question 4), near-vision activities (question 5-7), far-vision activities (question 8-10), peripheral vision (question 11), social function (question 12-13), color vision (question 14), driving (question 15-16), social role limitations (question 17-19), degree of dependence (question 20-22) and mental -health status (question 23-25). The total score of each subscale ranges from 0 (worst possible functioning) to 100 (best possible functioning). And, the final composite score of NEI-VFQ-25 is calculated by averaging the scores of all 11 subscales, except the general-health subscale.

#### 2.6.2 Headache impact test

Headache Impact Test (HIT-6) is a verified tool that had been commonly applied to measure the impact of headache on the daily function. The final score of HIT-6 is the summation of 6 items and ranges from 36 to 78 points, with a higher score reflecting a greater impact. According to the HIT-6 score, the severity of the impact of headache is categorized into 4 grades: little or no impact (≤ 49), moderate impact (50–55), substantial impact (56–59), and intense impact (60–78).

#### 2.6.3 Hamilton anxiety rating scale

Hamilton Anxiety Scale (HAMA) is one of the most commonly used scale in psychiatry for assessing anxiety, which is not only an important diagnostic tool for anxiety disorders, but also can classify the degree of anxiety. HAMA consists of 14 items and assesses the psychological and physical symptoms of anxiety in the recent one week. The scoring criteria for each item ranges from 0 (asymptomatic) to 4 (extremely severe symptoms) points. A total score of less than 7 indicates no anxiety, 7–14 may indicate anxiety, 14 or above definitely indicates anxiety, 14 to 17 indicates mild anxiety, 18 to 24 indicates moderate anxiety, and 25 to 30 indicates severe anxiety.

#### 2.6.4 Hamilton depression rating scale

Hamilton Depression Scale (HAMD) is one of the most commonly used scale in psychiatry to assess depressive states, with the advantages of simple operation and high reliability. HAMD has three versions: 17 items, 21 items and 24 items, of which the 24-item version was used in this study. According to the total score of HAMD, the severity of depression was classified into 4 grades: less than 7 indicates no depression, above 8 indicates depression, 8–20 indicates mild depression, 21–35 indicates moderate depression, and above 35 indicates severe depression.

#### 2.6.5 Health-related quality of life

Health-related quality of life (HR-QOL) is a commonly used tool for investigating patients’ health conditions. This study applied the German version of the self-report questionnaire SF-36, which has 36 questions and forms 8 subscales and 2 summary scores. HR-QOL-SF-36 consists of eight components including: physical function (PF), role-physical (RP), bodily pain (BP), general health perceptions (GH), vitality (VT), social function (SF), role-emotional (RE), and mental health (MH). The scoring range of each scale is from 0 to 100, with a higher score reflecting a higher degree of health. Moreover, two summary measures of physical condition score (PCS) and mental condition score (MCS) can be calculated based on the weighted scores of the 8 subscales. The standardized PCS and MCS have a mean value of 50 points and a standard deviation (SD) of 10 points, with higher scores indicating a better HR-QOL. One SD below mean value represents an impaired HR-QOL, two SD below mean value represents a severely impaired HR-QOL.

### 2.7 Data analysis

Data distribution was determined using the Kolmogorov–Smirnov test. Measurement variables were expressed as mean values ± standard deviations (M ± SD). The differences among four groups were analyzed by One-way ANOVA test with Bonferroni correction. Categorical variables were compared using chi-square test, Fisher’s exact test and Yates’ correction. Multiple linear regression analyses were used to investigate the correlation index of the possible influencing factors. Adjusted R² (A-R²) was used to evaluate the explanatory power of the regression model, and variance inflation factor (VIF) was used to perform collinearity diagnosis on multiple independent variables. SPSS version 18.0 software (SPSS Inc., USA) and GraphPad Prism 8 were used to perform the analysis, and two-tailed P<0.05 was considered statistically significant.

## 3 Results

### 3.1 Comparisons of clinical features and questionnaires outcomes of different QOL groups

A total of 94 PAs patients were enrolled in this study. Among them, 3 patients with cerebrospinal fluid leakage, 2 patients with intracranial infection, and 6 patients with pituitary dysfunction were excluded from the study due to surgical complications. At last, 83 PAs patients were recruited according to the inclusion and exclusion criteria, who accomplished the questionnaires preoperatively and at 1/3 months postoperatively. Those patients were further classified into simple physiological function impairment (PFI) (PCS<40, MCS>40) group (n=7), simple mental function impairment (MFI) (MCS<40, PCS>40) group (n=30), both physiological and mental function impairment (PMFI) (PCS<40, MCS<40) group (n=7), and neither physiological or mental function impairment (NPMFI) (MCS>40, PCS>40) group (n=39) according to the score of PCS and MCS preoperatively. The constitute of gender, tumor size, pathological type, hypopituitarism and vision/visual field impairment among the four groups had no significant differences. As for hypopituitarism, the most commonly affected pituitary gland axis was adrenal axis and thyroid axis, with 8 patients manifested as low cortisol level and 6 patients manifested as low free T4 and TSH. However, the age, the composite score of NEI-VFQ-25, and the grades of HIT-6, HAMA and HAMD had significant differences. Specifically, patients in the MFI group had the highest tendency towards anxiety and depression, patients in the PFI group had the lowest NEI-VFQ-25 score, and patients in the PMFI group were youngest and had the highest grades of HIT-6 among those four groups. In all, the age, vision/visual field impairment, headache, anxiety and depression might be correlated with the QOL of PAs patients preoperatively (**Table 1**).

**Table 1:** The differences of clinical features and questionnaires scores of four groups before ETSS.

Variable	MFI	PFI	PMFI	NPMFI	F/χ^2^	P
Age (M±SD, years)	43.0±15.8	54.6±10.0	40.9±15.9	55.8±16.4	3.445	**0.021** ^*^
Gender (n, %)					1.348	0.718
Male	2 (28.6%)	15 (50.0%)	3 (42.9%)	20 (51.3%)		
Female	5 (71.4%)	15 (50.0%)	4 (57.1%)	19 (48.7%)		
Tumor size (n, %)					4.233	0.645
Micro adenomas	0 (0.0%)	0 (0.0%)	1 (14.3%)	3 (7.7%)		
Macro adenomas	6 (85.7%)	24 (80.0%)	5 (71.4%)	28 (71.8%)		
Giant adenomas	1 (14.3%)	6 (20.0%)	1 (14.3%)	8 (20.5%)		
Pathological type (n, %)					8.784	0.889
TSH	0 (0.0%)	1 (3.3%)	0 (0.0%)	1 (2.6%)		
ACTH	1 (14.3%)	2 (6.7%)	1 (14.3%)	3 (7.7%)		
GTH	1 (14.3%)	12 (40.0%)	1 (14.3%)	18 (46.2%)		
PRL	3 (42.8%)	5 (16.7%)	1 (14.3%)	7 (17.8%)		
GH	1 (14.3%)	3 (10.0%)	1 (14.3%)	4 (10.3%)		
NFPA	1 (14.3%)	7 (23.3%)	3 (42.8%)	6 (15.4%)		
Vision/visual field (n, %)					0.711	0.871
With impairment	4 (57.1%)	21 (70.0%)	4 (57.1%)	26 (66.7%)		
Without impairment	3 (42.9%)	9 (30.0%)	3 (42.9%)	13 (33.3%)		
Hypopituitarism (n, %)					0.496	0.920
With	1 (14.3%)	6 (20.0%)	1 (14.3%)	9 (23.1%)		
Without	6 (85.7%)	24 (80.0%)	6 (85.7%)	30 (76.9%)		
NEI-VFQ-25 (M±SD)	87.5±16.2	78.5±15.6	81.9±16.1	88.6±10.2	3.466	**0.02** ^*^
HIT-6 (n, %)					19.938	**0.018** ^*^
No impact	5 (71.4%)	21 (70.0%)	3 (42.8%)	36 (92.3%)		
Moderate impact	1 (14.3%)	6 (20.0%)	2 (28.6%)	2 (5.1%)		
Substantial impact	0 (0.0%)	0 (0.0%)	1 (14.3%)	0 (0.0%)		
Intense impact	1 (14.3%)	3 (10.0%)	1 (14.3%)	1 (2.6%)		
HAMA (n, %)					8.449	**0.038** ^*^
No anxiety	4 (57.1%)	28 (93.3%)	6 (85.7%)	36 (92.3%)		
Possible anxiety	3 (42.9%)	2 (6.7%)	1 (14.3%)	3 (7.7%)		
Mild anxiety	0 (0.0%)	0 (0.0%)	0 (0.0%)	0 (0.0%)		
Moderate anxiety	0 (0.0%)	0 (0.0%)	0 (0.0%)	0 (0.0%)		
Severe anxiety	0 (0.0%)	0 (0.0%)	0 (0.0%)	0 (0.0%)		
HAMD (n, %)					20.436	**<0.001** ^*^
No depression	3 (42.9%)	28 (93.3%)	4 (57.1%)	37 (94.9%)		
Mild depression	4 (57.1%)	2 (6.7%)	3 (42.9%)	2 (5.1%)		
Moderate depression	0 (0.0%)	0 (0.0%)	0 (0.0%)	0 (0.0%)		
Severe depression	0 (0.0%)	0 (0.0%)	0 (0.0%)	0 (0.0%)		

MFI, mental function impairment; PFI, physiological function impairment; PMFI, physiological and mental function impairment; NPMFI, neither physiological nor mental function impairment; TSH, thyroid-stimulating hormone; ACTH, adrenocorticotropic hormone; GTH, gonadotropic hormone; PRL, prolactinoma; GH, growth hormone; NFPA, non-functional pituitary adenoma; NEI-VFQ, National Eye Institute Visual Function Questionnaire; HIT-6, Headache Impact Test-6; HAMA/HAMD, Hamilton Anxiety Scale/Hamilton Depression Scale; ETSS, endoscopic transsphenoidal surgery. P<0.05 represents statistically significant (marked with * and bold).

### 3.2 Correlations between questionnaires scores and QOL of patients with PAs before ETSS

In order to find out which clinical aspects could influence the QOL of patients with PAs before ETSS, multiple linear regressions were performed. The regressions on the questionnaires scores and PCS/MCS indicated that PCS was positively correlated with NEI-VFQ-25 composite score and negatively correlated with HIT-6 and HAMD scores, and MCS was positively correlated with age and negatively correlated with HIT-6, HAMA and HAMD scores (**Table 2**). The regressions on the twelve subscales of NEI-VFQ-25 scores and PCS/MCS indicated that PCS was positively correlated with general-health status, general vision, ocular pain, near-vision activities, far-vision activities, peripheral vision, social function, driving, social role limitations, degree of dependence and mental-health status scores, and MCS was positively correlated with general- health status, degree of dependence and mental-health status scores (**Table 3**). Furthermore, the regressions on the eight subscales of SF-36 scores and four questionnaires scores indicated that PF was negatively correlated with HIT-6, HAMA and HAMD scores, RP was positively correlated with NEI-VFQ-25 composite score and negatively correlated with HAMD score, BP and VT was negatively correlated with HIT-6, HAMA and HAMD scores, GH, SF, RE and MH was positively correlated with NEI-VFQ-25 composite score and negatively correlated with HIT-6, HAMA and HAMD scores (**Table 4**). In a word, those results indicated that vision/visual field impairment, headache, anxiety/depression emotion could influence the QOL of PAs patients preoperatively.

**Table 2:** Multiple linear regressions on the questionnaires scores and PCS/MCS before ETSS.

Variable	PCS (A-R²=0.172 F=4.398 P=0.001^*^)		MCS (A-R²=0.589 F=24.501 P<0.001^*^)	
	CI	P value	CI	P value
Age (VIF=1.126)	0.007	0.475	0.315	**0.002** ^*^
NEI-VFQ-25 (VIF=1.087)	0.307	**0.002** ^*^	0.123	0.134
HIT-6 (VIF=1.510)	-0.277	**0.006** ^*^	-0.377	**<0.001** ^*^
HAMA (VIF=2.467)	-0.107	0.168	-0.455	**<0.001** ^*^
HAMD (VIF=2.493)	-0.264	**0.008** ^*^	-0.747	**<0.001** ^*^

PCS, physical condition score; MCS, mental condition score; ETSS, endoscopic transsphenoidal surgery; NEI-VFQ-25, National Eye Institute Visual Function Questionnaire-25; HIT-6, Headache Impact Test-6; HAMA/HAMD, Hamilton Anxiety Scale/Hamilton Depression Scale; CI, correlation index; A-R^2^, adjusted R^2^; VIF, variance inflation factor. P<0.05 represents statistically significant (marked with * and bold).

**Table 3:** Multiple linear regressions on the NEI-VFQ-25 scores and PCS/MCS before ETSS.

Variable	PCS (A-R²=0.081 F=1.604 P=0.111)		MCS (A-R²=0.351 F=4.691 P<0.001^*^)	
	CI	P value	CI	P value
General-health status (VIF=1.256)	0.257	**0.010** ^*^	0.603	**<0.001** ^*^
General vision (VIF=2.370)	0.237	**0.015** ^*^	0.098	0.189
Ocular pain (VIF=2.203)	0.285	**0.004** ^*^	0.068	0.270
Near-vision activities (VIF=5.589)	0.279	**0.005** ^*^	-0.034	0.381
Far-vision activities (VIF=8.038)	0.228	**0.019** ^*^	0.050	0.326
Peripheral vision (VIF=6.372)	0.273	**0.006** ^*^	0.087	0.217
Social function (VIF=3.051)	0.190	**0.043** ^*^	0.172	0.061
Color vision (VIF=1.570)	0.012	0.456	0.068	0.272
Driving (VIF=1.373)	0.233	**0.017** ^*^	0.086	0.219
Social role limitations (VIF=2.890)	0.232	**0.017** ^*^	0.124	0.133
Degree of dependence (VIF=3.706)	0.229	**0.019** ^*^	0.211	**0.028** ^*^
Mental-health status (VIF=3.561)	0.256	**0.010** ^*^	0.197	**0.037** ^*^

NEI-VFQ-25, National Eye Institute Visual Function Questionnaire-25; PCS, physical condition score; MCS, mental condition score; ETSS, endoscopic transsphenoidal surgery; CI, correlation index; A-R^2^, adjusted R^2^; VIF, variance inflation factor. P<0.05 represents statistically significant (marked with * and bold).

**Table 4:** Multiple linear regression on the questionnaires scores and HR-QOL subscales before ETSS.

Variable	NEI-VFQ-25 (VIF=1.087)		HIT-6 (VIF=1.439)		HAMA (VIF=2.452)		HAMD (VIF=2.493)	
	CI	P value	CI	P value	CI	P value	CI	P value
PF (A-R²=0.160 F=4.897 P = 0.001 ^*^)	-0.014	0.449	-0.286	0.004^*^	-0.345	0.001^*^	-0.439	**<0.001** ^*^
RP (A-R²=0.413 F=15.420 P<0.001^*^)	0.469	<0.001^*^	-0.129	0.123	-0.123	0.135	-0.453	<0.001^*^
BP (A-R²=0.588 F=30.205 P<0.001^*^)	-0.034	0.381	-0.763	**<0.001** ^*^	-0.450	**<0.001** ^*^	-0.512	**<0.001** ^*^
GH (A-R²=0.364 F=12.755 P<0.001^*^)	0.187	**0.046** ^*^	-0.318	**0.002** ^*^	**-0.319**	**0.002** ^*^	-0.589	**<0.001** ^*^
VT (A-R²=0.454 F=18.031 P<0.001^*^)	0.105	0.173	-0.521	**<0.001** ^*^	**-0.503**	**<0.001** ^*^	-0.647	**<0.001** ^*^
SF (A-R²=0.450 F=17.766 P<0.001^*^)	0.234	**0.017** ^*^	-0.330	**0.001** ^*^	**-0.402**	**<0.001** ^*^	-0.659	**<0.001** ^*^
RE (A-R²=0.589 F=30.351 P<0.001^*^)	0.188	**0.044** ^*^	-0.320	**0.002** ^*^	**-0.386**	**<0.001** ^*^	-0.739	**<0.001** ^*^
MH (A-R²=0.373 F=13.188 P<0.001^*^)	0.054	**0.315**	-0.411	**<0.001** ^*^	**-0.425**	**<0.001** ^*^	-0.618	**<0.001** ^*^

HR-QOL, health-related quality of life; ETSS, endoscopic transsphenoidal surgery; NEI-VFQ-25, National Eye Institute Visual Function Questionnaire-25; HIT-6, Headache Impact Test-6; HAMA/HAMD, Hamilton Anxiety Scale/Hamilton Depression Scale; PF, physical function; RP, role-physical; BP, bodily pain; GH, general health perceptions; VT, vitality; SF, social function; RE, role-emotional; MH, mental health; CI, correlation index; A-R^2^, adjusted R^2^; VIF, variance inflation factor. P<0.05 represents statistically significant (marked with * and bold).

### 3.3 Correlations between questionnaires scores and QOL of patients with PAs after ETSS

Aiming to further analyze whether the alterations of vision/visual field impairment, headache, anxiety and depression emotion could influence the improvement of the QOL of PAs patients postoperatively, multiple linear regressions on the alteration of SF-36 scores and the alterations of NEI-VFQ-25 composite score, HIT-6, HAMA and HAMD scores were performed subsequently. The comparisons of the questionnaires and SF-36 scores among 3 time points including before ETSS, 1 month and 3 months after ETSS indicated that NEI-VFQ-25 composite score, PCS and MCS scores gradually increased, and HIT-6, HAMA and HAMD scores gradually decreased (**Table 5**). The regressions on the questionnaires scores and PCS/MCS improvement after ETSS indicated that both PCS and MCS improvement at 1 and 3 months after ETSS was positively correlated with the increased value of NEI-VFQ-25 composite score and the decreased value of HIT-6, HAMA and HAMD scores (**Table 6**). The regressions on the twelve subscales of NEI-VFQ-25 scores and PCS/MCS improvement indicated that the PCS improvement was positively correlated with the increased value of general-health status and general vision at 1 month after ETSS, the increased value of ocular pain, near-vision activities, far-vision activities, peripheral vision and degree of dependence at 1 and 3 months after ETSS, and the increased value of social function at 3 months after ETSS. And, the MCS improvement was positively correlated with the increased value of general-health status, peripheral vision, social function, social role limitations and degree of dependence at 1 and 3 months after ETSS, and the increased value of general vision and far-vision activities at 1 month after ETSS (**Table 7**). The regressions on the improvement of eight subscales of SF-36 scores and the alterations of four questionnaires scores indicated that the increased value of PF was positively correlated with the decreased value of HIT-6 at 3 months after ETSS and the decreased value of HAMA and HAMD at 1 and 3 months after ETSS, RP was positively correlated with the increased value of NEI-VFQ-25 at 1 and 3 months after ETSS and positively correlated with the decreased value of HIT-6 and HAMA at 1 month and the decreased value of HAMD at 1 and 3 months after ETSS, BP and MH was negatively correlated with the decreased value of HIT-6, HAMA and HAMD at 1 and 3 months after ETSS, and GH, VT, SF and RE was positively correlated with the increased value of NEI-VFQ-25 and positively correlated with the decreased value ofHIT-6, HAMA and HAMD at 1 and 3 months after ETSS (**Table 8**). Taking together, those results indicated that the improvement of vision/visual field impairment, and the remissions of headache, anxiety and depression emotion could beneficially influence the improvement of the QOL of PAs patients postoperatively.

**Table 5:** Comparisons of the questionnaires and HR-QOL scores among 3 time points around ETSS.

Variable	Before ETSS	1 month after ETSS	3 month after ETSS	F	P
PCS	43.9±14.8	50.4±12.6	55.4±8.0	18.884	**<0.001** ^*^
MCS	75.6±23.3	82.4±17.4	89.0±13.1	10.934	**<0.001** ^*^
NEI-VFQ-25	84.3±14.0	93.0±7.2	94.5±7.6	24.658	**<0.001** ^*^
HIT-6	41.8±8.5	39.3±7.2	36.7±2.2	12.488	**<0.001** ^*^
HAMA	3.3±2.5	2.1±3.0	0.9±1.4	19.964	**<0.001** ^*^
HAMD	4.1±3.5	2.6±4.7	1.3±2.2	12.266	**<0.001** ^*^

HR-QOL, health-related quality of life; ETSS, endoscopic transsphenoidal surgery; PCS, physical condition score; MCS, mental condition score; NEI-VFQ-25, National Eye Institute Visual Function Questionnaire-25; HIT-6, Headache Impact Test-6; HAMA/HAMD, Hamilton Anxiety Scale/Hamilton Depression Scale. P<0.05 represents statistically significant (marked with * and bold).

**Table 6:** Multiple linear regression on the questionnaires and PCS/MCS improvement after ETSS.

Variable	PCS(1-0)/(3-0) (A-R²=0.130 F=4.055 P=0.005^*^)/ (A-R²=0.075 F=2.666 P=0.038^*^)		MCS(1-0)/(3-0) (A-R²=0.384 F=13.767 P<0.001^*^)/ (A-R²=0.573 F=28.505 P<0.001^*^)	
	CI	P value	CI	P value
NEI-VFQ-25(1-0)/(3-0) (VIF=1.126/1.145)	0.257**/**0.255	**0.009** ^*^ **/0.010** ^*^	0.272/0.211	**0.006** ^*^ **/0.028** ^*^
HIT-6(1-0)/(3-0) (VIF=1.211/1.261)	-0.297/-0.220	**0.003** ^*^ **/0.023** ^*^	-0.324/-0.305	**0.001** ^*^ **/0.002** ^*^
HAMA(1-0)/(3-0) (VIF=3.978/2.521)	-0.258/-0.123	0.009^*^/0.133	-0.433/-0.418	**<0.001** ^*^ **/** **<0.001** ^*^
HAMD(1-0)/(3-0) (VIF=4.301/2.829 )	-0.323/-0.186	**0.001** ^*^ **/0.046** ^*^	-0.607/-0.736	**<0.001** ^*^ **/** **<0.001** ^*^

PCS, physical condition score; MCS, mental condition score; ETSS, endoscopic transsphenoidal surgery; NEI-VFQ-25, National Eye Institute Visual Function Questionnaire-25; HIT-6, Headache Impact Test-6; HAMA/HAMD, Hamilton Anxiety Scale/Hamilton Depression Scale; (1-0)/(3-0) The absolute difference value of 1 month after ETSS and before ETSS or the absolute difference value of 3 month after ETSS and before ETSS,CI Correlation index, A-R² Adjusted R², VIF Variance Inflation Factor. P<0.05 represents statistically significant (marked with * and bold).

**Table 7:** Multiple linear regressions on the NEI-VFQ-25 score and PCS/MCS improvement after ETSS.

Variable	PCS(1-0)/(3-0) (A-R²=0.009 F=1.064 P=0.402)/ (A-R²=0.014 F=1.105 P=0.370)		MCS(1-0)/(3-0) (A-R²=0.281 F=3.914 P<0.001^*^)/ (A-R²=0.288 F=4.009 P<0.001^*^)	
	CI	P value	CI	P value
General-health status(1-0)/(3-0) (VIF=1.336/1.275)	0.212/0.107	0.027^*^/0.167	0.572/0.570	**<0.001** ^*^ **/<0.001** ^*^
General vision(1-0)/(3-0) (VIF=2.870/2.325)	0.215/0.138	0.025^*^/0.107	0.241/0.163	0.014^*^/0.071
Ocular pain(1-0)/(3-0) (VIF=2.385/2.719)	0.198/0.237	**0.036** ^*^ **/0.016** ^*^	0.171/0.155	0.062/0.081
Near-vision activities(1-0)/(3-0) (VIF=4.350/4.160)	0.250/0.235	**0.011** ^*^ **/0.016** ^*^	0.156/0.093	0.079/0.202
Far-vision activities(1-0)/(3-0) (VIF=6.114/6.336)	0.220/0.187	**0.023** ^*^ **/0.045** ^*^	0.229/0.139	0.019^*^/0.105
Peripheral vision(1-0)/(3-0) (VIF=4.833/5.792)	0.221/0.275	**0.022** ^*^ **/0.006** ^*^	0.187/0.183	**0.045** ^*^ **/0.049** ^*^
Social function(1-0)/(3-0) (VIF=2.654/2.791)	0.123/0.210	0.133/0.028^*^	0.244/0.229	**0.013** ^*^ **/0.019** ^*^
Color vision(1-0)/(3-0) (VIF=1.005/1.009)	-0.083/-0.123	0.227/0.134	0.053/0.042	0.317/0.352
Driving(1-0)/(3-0) ( / )	/	**/**	/	**/**
Social role limitations(1-0)/(3-0) (VIF=2.430/2.301)	0.127/0.155	0.127/0.082	0.245/0.187	**0.013** ^*^ **/0.045** ^*^
Degree of dependence(1-0)/(3-0) (VIF=2.900/2.323)	0.269/0.244	**0.007** ^*^ **/0.013** ^*^	0.255/0.192	**0.010** ^*^ **/0.041** ^*^
Mental-health status(1-0)/(3-0) (VIF=2.030/1.793)	0.174/0.066	0.058/0.277	0.199/0.165	0.036^*^/0.068

NEI-VFQ-25, National Eye Institute Visual Function Questionnaire-25; PCS, physical condition score; MCS, mental condition score; ETSS, endoscopic transsphenoidal surgery; (1-0)/(3-0) The absolute difference value of 1 month after ETSS and before ETSS or the absolute difference value of 3 month after ETSS and before ETSS, CI Correlation index, A-R² Adjusted R², VIF Variance Inflation Factor. P<0.05 represents statistically significant (marked with* and bold).

**Table 8:** Multiple linear regressions on the questionnaires and HR-QOL subscales improvement after ETSS.

Variable	NEI-VFQ-25(1-0)/(3-0) (VIF=1.126/1.145)		HIT-6(1-0)/(3-0) (VIF=1.211/1.261)		HAMA(1-0)/(3-0) (VIF=3.978/2.521)		HAMD(1-0)/(3-0) (VIF=4.301/2.829)	
	CI	P value	CI	P value	CI	P value	CI	P value
PF(1-0)/(3-0) (A-R²=0.297 F=9.667 P<0.001^*^)/ (A-R²=0.113 F=3.609 P=0.009^*^)	0.116/ 0.035	0.149/ 0.378	-0.303/ -0.137	0.003^*^/ 0.108	-0.481/ -0.221	<0.001^*^/ 0.022^*^	-0.569/ -0.372	**<0.001** ^*^ **/<0.001** ^*^
RP(1-0)/(3-0) (A-R²=0.319 F=10.593 P<0.001^*^)/ (A-R²=0.320 F=10.663 P<0.001^*^)	0.429/ 0.411	<0.001^*^/<0.001^*^	-0.210/ -0.142	0.028^*^/ 0.101	-0.306/ -0.170	0.002^*^/ 0.062	-0.482/ -0.459	<0.001^*^/<0.001^*^
BP(1-0)/(3-0) (A-R²=0.500 F=21.528 P<0.001^*^)/ (A-R²=0.533 F=24.416 P<0.001^*^)	0.112/ 0.044	0.156/ 0.348	-0.701/-0.716	<0.001^*^/<0.001^*^	-0.413/ -0.447	<0.001^*^/<0.001^*^	-0.452/ -0.476	<0.001^*^/<0.001^*^
GH(1-0)/(3-0) (A-R²=0.299 F=9.725 P<0.001^*^)/ (A-R²=0.335 F=11.340 P<0.001^*^)	0.354/ 0.287	0.001^*^/ 0.004^*^	-0.262/-0.205	0.008^*^/ 0.032^*^	-0.346/ -0.337	0.001^*^/ 0.001^*^	-0.508/ -0.574	<0.001^*^/<0.001^*^
VT(1-0)/(3-0) (A-R²=0.401 F=14.724 P<0.001^*^)/ (A-R²=0.435 F=16.808 P=0.111)	0.280/ 0.229	0.005^*^/ 0.019^*^	-0.505/-0.396	<0.001^*^/<0.001^*^	-0.488/ -0.481	<0.001^*^/<0.001^*^	-0.563/ -0.661	<0.001^*^/<0.001^*^
SF(1-0)/(3-0) (A-R²=0.415 F=15.543 P<0.001^*^)/ (A-R²=0.455 F=18.124 P<0.001^*^)	0.353/ 0.289	0.001^*^/ 0.004^*^	-0.386/-0.266	<0.001^*^/0.007^*^	-0.419/ -0.376	<0.001^*^/<0.001^*^	-0.594/ -0.659	<0.001^*^/<0.001^*^
RE(1-0)/(3-0) (A-R²=0.439 F=17.033 P<0.001^*^)/ (A-R²=0.604 F=32.250 P<0.001^*^)	0.251/ 0.223	0.011^*^/ 0.021^*^	-0.234/-0.292	0.017^*^/ 0.004^*^	-0.421/ -0.383	<0.001^*^/<0.001^*^	-0.637/ -0.737	<0.001^*^/<0.001^*^
MH(1-0)/(3-0) (A-R²=0.312 F=10.298 P<0.001^*^)/ (A-R²=0.369 F=12.978 P<0.001^*^)	0.274/ 0.168	**0.006**/ 0.065	-0.413/-0.295	<0.001^*^/ 0.003^*^	-0.426/ -0.357	<0.001^*^/<0.001^*^	-0.524/ -0.606	<0.001^*^/<0.001^*^

HR-QOL, health-related quality of life; ETSS, endoscopic transsphenoidal surgery; NEI-VFQ-25, National Eye Institute Visual Function Questionnaire-25; HIT-6, Headache Impact Test-6; HAMA/HAMD, Hamilton Anxiety Scale/Hamilton Depression Scale; PF, physical function; RP, role-physical; BP, bodily pain; GH, general health perceptions; VT, vitality; SF, social function; RE, role-emotional; MH, mental health; (1-0)/(3-0) The absolute difference value of 1 month after ETSS and before ETSS or the absolute difference value of 3 month after ETSS and before ETSS, CI Correlation index, A-R² Adjusted R², VIF Variance Inflation Factor. P<0.05 represents statistically significant (marked with * and bold).

## 4 Discussion

It is well known that ETSS not only can precisely and minimal invasively accomplish the resection of PAs, but also can relieve the compression effect on the nerves and blood vessels adjacent to the tumor and restore the normal function of pituitary gland. However, the extent of the improvement of PAs patients’ QOL before and after ETSS and the key influencing factors related to it apart from operations have not been fully clarified. Accordingly, we conducted this prospective and observational study to analyze the changes of QOL in patients with PAs before and after ETSS, and analyzed the key factors related to the improvement of the QOL of PAs by conducting questionnaires survey related to vision, headache, anxiety and depression. It was found that the vision/visual field impairment, headache, anxiety/depression emotion not only could influence the QOL of PAs patients preoperatively, but also could influence the improvement of the QOL of PAs patients postoperatively. Those outcomes remind us the importance of the concern and treatment on the vision/visual field impairment, headache, anxiety/depression emotion of PAs.

The QOL of PAs patients is mostly impaired to some degrees by various related factors, which have been studied by several researchers, though no consensus have been formulated (17, 18). In this study, patients with PAs were first divided into solely PCS impaired (PFI) group, solely MCS impaired (MFI) group, both PCS and MCS impaired (PMFI) group, and neither PCS or MCS impaired (NPMFI) group according to the physiological dimension (PCS) score and psychological dimension (MCS) score. Subsequently, we compared the differences among the four groups in terms of age, gender, tumor size, pathological type, presence/absence of visual field impairment, with/without hypopituitarism, and the scores of NEI-VFQ-25, HIT-6, HAMA/HAMD. We found that there were no significant differences among the four groups in terms of gender, tumor size, pathological type, hypopituitarism and visual acuity/visual field impairment, but there were significant differences in terms of age and the scores of NEI-VFQ-25, HIT-6, HAMA and HAMD. There into, patients with PAs in MFI group had the highest tendency of anxiety and depression, in PFI group had the lowest NEI-VFQ-25 score, and in PMFI group were the youngest and had the highest HIT 6 score. Previous studies have reported that the impairment of HR-QOL in PAs patients is correlated with tumor size, endocrine/pathological types, which is inconsistent with the results of our study (18, 19). The possible reason is that the tumor size of most of the recruited patients in this study were large adenomas of 1-3cm, while huge adenomas larger than 3cm were relatively rare. Furthermore, there were no significant differences in the distribution of objective visual field examination results, but there was a significant difference in the NEI-VFQ-25 composite score among the four groups. These contradictory results indicated that the subjective feelings about visual and field impairment were different among patients, with poorer subjective feelings leading to worse QOL, especially in the psychological dimension (9). Corresponding to this, there were also significant differences in the HAMA/HAMD scores among four groups, with higher scores indicated a higher tendency towards anxiety/depression and were more prone to have impaired QOL in the psychological dimension. In addition, previous studies have suggested that hypopituitarism is also one of the important factors affecting the QOL of patients with PAs (4, 13). Hypopituitarism is caused by the compression of normal pituitary tissue by pituitary tumors, especially large adenomas, whose core symptom is the dysfunction of the target gland axis, and the onset is insidious and is often mistaken for “sub-health”. The main clinical manifestations include fatigue, weakness, depression, loss of appetite or low libido. Subsequently, the patient may experience difficulties in performing daily life or work, memory decline, hard concentrating or sexual dysfunction, which lead to a decline in QOL eventually. However, our study found that the hypopituitarism seemed to have no correlation with the QOL of PAs patients preoperatively. The possible reason for this inconformity is that the tumor size of the included PAs patients was mostly 1-3cm, and the probability of hypopituitarism was relatively low. In all, these results indicated that the preoperative QOL of PAs patients was correlated with age, visual field perception, headache perception and anxiety and depression tendency, which was a focus worthy of clinicians’ attention before ETSS.

The compressive effects of PAs on the optic nerve and optic chiasm can cause vision-related symptoms such as visual field defects, vision decline or eye paralysis. Endoscopic surgical resection of PAs can improve vision to some degrees in 80%-90% of patients (9, 20). These improvements can manifest within a few days after the ETSS and continue to improve in the following weeks, but the exact time frame remains unclear (9, 20). Previous studies have also reported VR-QOL in patients with vision impairment caused by several diseases including PAs, glaucoma, ischemic injury and so on (20, 21). However, few studies have directly investigated the imaging of VR-QOL in patients with PAs and its correlation with HR-QOL after the ETSS surgery. In this study, the NEI-VFQ-25 was used to evaluate the changes of VR-QOL in patients with PAs before ETSS, one/three months after ETSS, and analyzed its correlation with the improvement of HR-QOL after ETSS. We found that the composite scores of NEI-VFQ-25 were positively correlated with the preoperative physiological dimension of QOL (PCS), but were not correlated with the psychological dimension of QOL (MCS). Moreover, the scores of the 12 dimensions of NEI-VFQ-25 were all positively correlated with PCS, and the composite score of NEI-VFQ-25 was also positively correlated with RP, GH, SF, RE and MH among the 8 dimensions of HR-QOL. The composite score and the 12 dimensions scores of NEI-VFQ-25 gradually increased at 1 month and 3 months after ETSS, which were positively correlated with the improvement of HR-QOL. Unlike the situations before ETSS, the increased values of the composite score and 12 dimensions scores of NEI-VFQ-25 at 1 month and 3 months after ETSS were not only positively correlated with the increased value of PCS, but also positively correlated with the increased value of MCS, indicating the improvement of postoperative visual acuity played a key role in the improvement of both physical and psychological dimensions of QOL. Furthermore, the increased value of the NEI-VFQ-25 composite score was also positively correlated with the increased value of RP, GH, VT, SF, RE and MH of HR-QOL. Above results suggested that patients with PAs who underwent ETSS acquired improvement in multiple dimensions of vision including general vision, close range activities, mental health, role disorders, dependence, peripheral vision and driving, and these improvements further played key roles in enhancing the QOL of patients with PAs. Interestingly, this study also found that there was a correlation between the improvement of VR-QOL and the improvement of MCS after ETSS, suggesting the improvement of postoperative vision promoted the improvement of mental health. This phenomenon might be related to the fact that PAs patients accompanied by visual impairment were worried about the possibility of continued decline of vision before surgery, which in turn leaded to restricted activity and less working competence (9). And, these negative psychological states were adjusted by PAs patients themselves after ETSS as their vision gradually improved. Moreover, we found the improvement amplitude of the composite score of NEI-VFQ-25 in PAs patients was significantly greater at 1 month than at 3 months after ETSS, suggesting the early postoperative vision improvement was crucial for the improvement of VR-QOL/HR-QOL. In a word, these results suggested that the adequate decompression and meticulous protection of optic nerve during ETSS, as well as the continuous promotion measures for visual recovery after ETSS are of great significance for improving the QOL of PAs patients.

PAs can squeeze the brain tissue around seller region and cause intermittent or persistent headache, which is an initial symptom of patients to seek medical treatment (21, 22). Headache can affect a patient’s work, life and psychological mood, thereby damaging the QOL. Some researchers analyzed the impact of different degrees of headache on the QOL of PAs patients and found it was significantly correlated with the psychological dimension of QOL, and ETSS could improve the postoperative QOL of patients by alleviating headache symptoms (22, 23). This study found that the HIT-6 score was negatively correlated with both the physiological dimension (PCS) and the psychological dimension (MCS) of preoperative HR-QOL, and was negatively correlated with PF, BP, GH, VT, SF, RE and MH of HR-QOL as well, suggesting headache had a negative impact on all aspects of a patient’s QOL. The HIT-6 score gradually decreased at 1 month and 3 months after ETSS, and the decreased value was positively correlated with the improvement of HR-QOL. Similar to the situation before ETSS, the decreased values of HIT-6 at 1 month and 3 months after ETSS not only were positively correlated with the increased values of PCS/MCS, but also were positively correlated with the increased values of PF, BP, GH, VT, SF, RE and MH of HR-QOL. These results indicated that headache not only affected the physiological functions of patients but also influenced their psychological emotions, and the relief of postoperative headache played a key role in the improvement of both physical and psychological dimensions of QOL.

In addition to above mentioned factors including visual field defects, vision decline, headache and endocrine changes, some patients might develop anxiety and depression emotion, which could damage the QOL of PAs patients as well (8, 24–26). Therefore, this study also analyzed the correlation between the HAMA/HAMD scores and the QOL of patients with PAs before and after ETSS. Before ETSS, the HAMA score was only negatively correlated with the psychological dimension of QOL (MCS), while the HAMD score was negatively correlated with both the physiological and the psychological dimension of QOL (PCS and MCS). Moreover, the HAMA and HAMD scores were negatively correlated with PF, BP, GH, VT, SF, RE and MH of QOL, suggesting the anxiety and depression emotion had a negative impact on all aspects of the QOL of PAs patients. Similar to the situation before ETSS, the decreased values of HAMA and HAMD scores at 1 month and 3 months after ETSS were not only positively correlated with the increased values of PCS and MCS, but also were positively correlated with the increased values of PF, BP, GH, VT, SF, RE and MH of HR-QOL. These results indicated that the relief of postoperative anxiety and depression emotion after ETSS played a role in improving both the physical and psychological aspects of HR-QOL.

In conclusion, this prospective and observational study comprehensively analyzed the potential factors that might influence the HR-QOL in patients with PAs before and after ETSS. It was found that the impaired HR-QOL of patients with PAs before ETSS was correlated with headache, subjective perception of vision and visual field impairment, and the anxiety/depression emotions. The improvement of HR-QOL after ETSS was closely related to the relief of headache, the improvement of subjective perception of vision and visual field impairment, and the relief of anxiety and depression emotions. These results suggested that we not only should focus on the surgical techniques of ETSS, but also should pay attention to the identification and treatment of the subjective symptoms of patients with PAs during the preoperative period and 1–3 months after ETSS in clinical practice, which is of great significance for improving the QOL of these patients.

## Data Availability

The raw data supporting the conclusions of this article will be made available by the authors, without undue reservation.

## References

[1] Ostrom QT Patil N Cioffi G Waite K Kruchko C Barnholtz-Sloan JS . CBTRUS statistical report: Primary brain and other central nervous system tumors diagnosed in the United States in 2013-2017. Neuro-Oncology. (2020) 22:iv1–iv96. doi: 10.1093/neuonc/noaa200 33123732 PMC7596247

[2] Tritos NA Miller KK . Diagnosis and management of pituitary adenomas: A review. JAMA J Am Med Assoc. (2023) 329:1386–98. doi: 10.1001/jama.2023.5444 37097352

[3] Carmel Neiderman NN Kaufman S Bilaus R Wengier A Ziv Baran T Abergel A . Long-term quality of life among patients undergoing endoscopic pituitary gland surgery. J Clin Med. (2024) 13(21):6371. doi: 10.3390/jcm13216371 39518511 PMC11546924

[4] Pala A Grübel N Mayer B Becker R Sommer F Schmitz B . Endocrine outcome and quality of life after trans -sphenoidal resection of pituitary adenoma-a prospective randomized single-blinded study comparing endoscopic versus microscopic resection. Neurol Int. (2025) 17(1):5. doi: 10.3390/neurolint17010005 39852769 PMC11767373

[5] de Almeida JR Hueniken K Xie M Monteiro E Zadeh G Kalyvas A . Multi-institutional comparison of quality of life between open versus endoscopic skull base approaches. Laryngoscope Investig Otolaryngol. (2025) 10:e70082. doi: 10.1002/lio2.70082 39840025 PMC11748208

[6] Forsyth PA Posner JB . Headaches in patients with brain tumors: A study of 111 patients. Neurology. (1993) 43:1678–83. doi: 10.1212/wnl.43.9.1678 8414011

[7] Gondim JA de Almeida JP de Albuquerque LA Schops M Gomes E Ferraz T . Headache associated with pituitary tumors. J Headache Pain. (2008) 10:15–20. doi: 10.1007/s10194-008-0084-0 19067118 PMC3451766

[8] Wolf A Coros A Bierer J Goncalves S Cooper P Van Uum S . Quantitative evaluation of headache severity before and after endoscopic transsphenoidal surgery for pituitary adenoma. J Neurosurg. (2015) 124:1627–33. doi: 10.3171/2015.5.JNS1576 26495954

[9] Wolf A Coros A Bierer J Goncalves S Cooper P Van Uum S . Quantitative evaluation of vision-related and health-related quality of life after endoscopic transsphenoidal surgery for pituitary adenoma. J Neurosurg. (2016) 127:409–16. doi: 10.3171/2016.7.JNS16200 27715435

[10] Ishikawa T Takeuchi K Nagatani T Aimi Y Tanemura E Tambara M . Quality of life changes before and after transsphenoidal surgery for sellar and parasellar lesions. World Neurosurg. (2018) 122:e1202–10. doi: 10.1016/j.wneu.2018.11.017 30447458

[11] Crespo I Santos A Resmini E Valassi E Martínez-Momblán MA Webb SM . Improving quality of life in patients with pituitary tumours. Eur Endocrinol. (2013) 9:32–6. doi: 10.17925/EE.2013.09.01.32 30349608 PMC6193521

[12] Sagan KP Andrysiak-Mamos E Tyburski E Sagan LM Syrenicz A . Quality of life and sleep in patients with pituitary adenoma in relation to tumor type and compression of the optic chiasm. J Clin Med. (2021) 10(9):1879. doi: 10.3390/jcm10091879 33926090 PMC8123647

[13] Xie S Shao Z Shao D Zheng X Tang H Li Y . Correlation between intrasellar pressure, pituitary adenoma invasiveness, pituitary dysfunction, and apoplexy. Front Endocrinol. (2025) 16:1700711. doi: 10.3389/fendo.2025.1700711 41480352 PMC12753394

[14] Rutland JW Gill CM Ladner T Goldrich D Villavisanis DF Devarajan A . Surgical outcomes in patients with endoscopic versus transcranial approach for skull base Malignancies: A 10-year institutional experience. Brit J Neurosurg. (2020) 36:79–85. doi: 10.1080/02688697.2020.1779659 32538686

[15] Schaberg MR . Quality of life outcomes after endoscopic approaches to intracranial tumors. Curr Opin Otolaryngo. (2018) 26:58–64. doi: 10.1097/MOO.0000000000000427 29210717

[16] Lillehei KO Travers S Barkhoudarian G Oyesiku NM Germano IM Ormond DR . Congress of neurological surgeons systematic review and evidence-based guidelines for the role of surgery for patients with functioning pituitary adenomas. Neurosurgery. (2025) 97:S24–35. doi: 10.1227/neu.0000000000003559 40815132

[17] Joustra GE van Rhee NF den Heijer MC Korsten-Meijer AGW Feijen RA Halmos GB . Very long-term follow-up of multidimensional health-related quality of life after endoscopic endonasal surgery for pituitary adenomas: A prospective cohort study. Head Neck. (2026) 48(4):1088–1096. doi: 10.1002/hed.70103 41312616 PMC12972636

[18] Castle-Kirszbaum M Wang YY King J Kam J Goldschlager T . Quality of life and surgical outcomes in incidental pituitary adenomas undergoing endoscopic endonasal resection. J Neurosurg. (2022) 138:567–73. doi: 10.3171/2022.5.JNS2286 35901767

[19] Ferreli F Lasagna C Canali L Baram A Bono BC Tropeano MP . A randomized prospective comparative study on sinonasal morbidity and quality of life of transsphenoidal endoscopic surgery for pituitary adenomas: Endonasal versus trans-septal approach. Eur Arch Oto-Rhino-L. (2023) 281:257–66. doi: 10.1007/s00405-023-08216-1 37673831

[20] Ulitin AIu Oliushin VIu Zhinzhina IV Ovcharenko OIu Getmanova VV Markova NV . Ophthalmological symptoms in patients with giant pituitary adenomas. Vestn Oftalmol. (2007) 123:36–42. 17672094

[21] Gall C Franke GH Sabel BA . Vision-related quality of life in first stroke patients with homonymous visual field defects. Health Qual Life Outcomes. (2010) 8:33. doi: 10.1186/1477-7525-8-33 20346125 PMC2859371

[22] Scaf CR Altoé A Ventura N Vincent M Kasuki L Gadelha MR . Headache in pituitary adenomas: Frequency, characteristics and outcome after treatment. Pituitary. (2025) 28:31. doi: 10.1007/s11102-025-01504-5 39924607

[23] Delport R King J Castle-Kirszbaum M Goldschlager T Caputo C Wang YY . Headache improvement following endoscopic resection of pituitary adenomas. World Neurosurg. (2023) 176:e456–61. doi: 10.1016/j.wneu.2023.05.082 37277024

[24] Yang G Shen S Zhang J Gu Y . Analysis of perioperative negative emotion risk factors in patients with pituitary adenoma and its impact on prognosis: A retrospective study. Gland Surg. (2022) 11:1240–50. doi: 10.21037/gs-22-387 35935570 PMC9346215

[25] Kalasauskas D Ernst A Mireri S Keric N Thavarajasingam SG Omran W . Psychological burden in patients with sellar masses under conservative and surgical management. Neurosurg Rev. (2025) 48:104. doi: 10.1007/s10143-025-03240-7 39883209 PMC11782327

[26] Zhang J Xu X Wang Z Jiang S . The effects of cognitive bias modification for attention and interpretation on the postoperative psychological resilience and quality of life of patients with pituitary adenoma: A randomized trial. Ann Palliat Med. (2021) 10:5729–37. doi: 10.21037/apm-21-782 33977745

